# Overall survival and prognostic factors prostate cancer in Kurdistan Province-Iran: a population-based study (2011-2018)

**DOI:** 10.1186/s12885-021-09078-8

**Published:** 2021-12-08

**Authors:** Mohammad Aziz Rasouli, Ghobad Moradi, Bushra Zareie, Heshmatollah Sofimajidpour, Sima Tozandehjani, Hedyeh Zafari, Fatemeh Gholami, Sonia Shahsavari, Parisa Hassani, Mahshid Mohammadian

**Affiliations:** 1 Social Determinants of Health Research Center, Research Institute for Health Development, Kurdistan University of Medical Sciences, Sanandaj, Iran; 2 Department of Epidemiology and Biostatistics, Faculty of Medicine, Kurdistan University of Medical Sciences, Sanandaj, Iran; 3 Department of Epidemiology, School of Public Health, Hamadan University of Medical Sciences, Hamadan, Iran; 4 Department of Urology, Faculty of Medicine, Kurdistan University of Medical Sciences, Sanandaj, Iran; 5 Department of Biological Sciences, Faculty of Science, University of Kurdistan, Sanandaj, Iran; 6 Department of Epidemiology, School of Public Health, Iran University of Medical Sciences, Tehran, Iran; 7 Department of Clinical Biochemistry, School of Medicine, Urmia University of Medical Sciences, Urmia, Iran

**Keywords:** Survival, Prostate Cancer, Population-Based Study, Kurdistan, Iran

## Abstract

**Background:**

The population-based survival rate is affected by the quality and effectiveness of health care systems. Overall, the survival of prostate cancer (PC) patients has improved over the past two decades worldwide. This study aimed to determine the overall survival rate and correlate it with the prognostic factors in patients with PC diagnosed in Kurdistan province.

**Methods:**

In a retrospective cohort study, 410 PC patients registered in Kurdistan province population-based cancer registry from March 2011 to 2018 were recruited. Kaplan–Meier method and log-rank test were used to analyze the overall survival rates of PC patients. A Multivariate Cox regression model was used to determine adjusted hazard ratios for different variables.

**Results:**

Of 410 patients with PC, 263 (64.1%) died within seven years due to the disease. The 1, 3, and 5 years survival rates were 93, 64.1, and 40.7%, respectively. According to the results of multiple Cox regression, the following factors were significantly related to PC survival: age at diagnosis (≥81-years old) (HR=2.23, 95% CI: 1.23-4.42) and 71-80 years old was (HR=1.26, 95% CI: 1.12-2.31), occupation (employee) (HR=0.42, 95% CI: 0.20–0.87), educational level: academic (HR=0.78, 95% CI: 0.64–0.91), AJCC stage of disease (HR=2.18, 95% CI: 1.9–3.68), Gleason score ≥ 9 (HR=7.12, 95% CI: 5.35–10.28), and Gleason score= 8 (HR=4.16, 95% CI: 2.50–6.93). There was less mortality rate among the patients who had received active care, radical prostatectomy, radiotherapy, combined treatment, and orchiectomy had a lower mortality rate than those who received no treatment (*P*<0.05).

**Conclusions:**

This study demonstrated that factors such as age at diagnosis, level of education, occupation, AJCC stage of disease, Gleason score, and type of treatments were influential factors in the survival of PC patients in Kurdistan province and needed more attention.

## Introduction

Prostate cancer (PC) is the second most prevalent cancer and the cause of the sixth cancer-caused death in men worldwide [1]. The prevalence and mortality rate of the disease is not similar between countries, and 75% of the cases happen at the age above 65 years old [2–4]. Cancer is the third most prevalent cancer in men and the sixth most prevalent cancer in Iran, so that it constitutes 7-9% of total cancer cases [5–7].

The prevalence of PC is not the same between different countries and races [8–10]. The difference is due to different reasons such as genetic capacity, exposure to the unknown environmental risk factor, differences in health care models, socioeconomic factors, cancer registration system, etc. [8–10]. Studies on cancers and determining probability and survival distribution of cancer patients based on demographical and clinical variables of patients are critical. To this end, survival analysis models can be used. Considering that long-term survival following prostate cancer treatment is widespread, it is crucial to estimate the survival rate and prepare the ground to meet the unique requirements of these patients [11–13].

Studies on estimating PC survival are growing. However, despite the ever-increasing recognition of the long-term outcomes of prostate cancer diagnosis, research works on this field are still disorganized [13, 14]. In Iran, the survival rate of PC is mainly based on the information of hospital files, so the five-year survival rate, according to some studies, is about 36% [15]. The survival rate based on population is affected by the quality and effectiveness of health care systems. In general, the survival rate of PC patients has been growing over the past few decades, especially in European countries [16, 17]. However, this progress is not compatible between countries, races, and socioeconomic groups. A deprivation gap was observed among patients with prostate cancer diagnosed in Scotland during 1996-2000 [17]..

The aim of assessing survival is to determine its effect on the patient, health system, and clinical care policies gap in post-treatment; Estimating resources to help patients achieve optimal health; increasing their quality of life, and survival after treatment is a significant issue of general health services.

### Materials and methods

In this retrospective cohort study, 410 patients with PC were collected from the cancer registry system in the Kurdistan University of Medical Sciences from 2011 to 2018. Kurdistan is a province located in the western region of Iran and constitutes eight counties. The majority of the residents of this province are farmers and ranchers. In the national census in 2016, the overall population of this province was estimated as 1,603,000, with 71% of them lived in urban areas, and 17.5% of them were over 50 years old. Most of the residents of the province are of Kurdish ethnicity.

The study was conducted from March 20, 2011, to March 19, 2018, in Kurdistan based on a primary diagnosis of histopathology and cancer registration system. In addition, the study was a population-based work. Diagnosis and registration were based on the International Categorization of Diseases (ICD10) and anatomic position of prostate cancer (C84). The required data were collected from Kurdistan Province Cancer Registration System. Data gathering was done in September 2019 (cutoff date).

The primary source data of PC patients were obtained from the cancer registry system. Other required data were collected from patients’ medical records, pathology reports, and the death system. Trained interviewers conducted an additional telephone survey to collect data, including survival status, age (at diagnosis), sex, occupation, level of education, marital status, place of residence, smoking and alcohol history, health status at the time of referring to the hospital, the date of death, and family history of PC. Pathologic data, including patients' information, included local tumor, Gleason score, and type of treatment (based on the medical and pathological report).

Survival time was measured from the diagnosis to death or the last follow-up. The subjects were studied in terms of age at diagnosis (≤60-years, 61-70 years, 71-80 years, and ≥81-years old), occupation (unemployed/retired, worker, self-employed, employee), education level (college, high school, junior high school, illiterate) marital status, and domicile. Based on the Gleason score, the tumors were categorized as low (≤6), moderate [7, 8], and high (≥9). Based on AJCC measures, the tumor stage was categorized as I, II, III, and IV, and the type of treatment included active surveillance, radical prostatectomy, radiotherapy, radiotherapy plus radical prostatectomy, orchiectomy, androgen therapy, and no treatment.

### Data analyses

The 1, 3, and 5 years’ survival rate and median of survival were investigated based on the variables under study. The difference in survival rate was measured for the subgroups using the log-rank test. Using the Kaplan-Meier method, overall survival, age at diagnosis, Gleason score, tumor stage, and type of treatment were demonstrated on a curve.

Univariable and multivariable regression Cox proportional hazard models were executed. To select and shrink the selected predictors according to their relative contribution to the final model, a least absolute shrinkage and selection operator (LASSO) method was performed [18]. For inclusion in a multivariate model, all those variables which had a p-value of less than 0.10 or were previously well-known confounders in such analysis were included in multivariate analysis in a stepwise approach. Internal validation was used by the bootstrap method, in which new datasets are created by random drawing from the sample with replacement [19]. The whole modeling process, i.e., developing a Cox regression model with a LASSO penalty, was reiterated in each of these new datasets.

The assumptions of the hazard proportionality have been tested by graphical methods (log (s) *t* vs. time) and Shoenfield residuals ph test [20]. There were no violations of the proportionality assumption for any of the covariates included in the PC-specific models. *P* < 0.05 was considered statistically significant. All statistical analyses were performed using Stata16.0 software (StataCorp, College Station, TX).

## Results

Of 410 patients with PC, 263 (64.1%) died within seven years due to the disease. The age at diagnosis was 68.3±8.24 years, and 56 individuals (13.7%) were younger than 60 years. The majority were city dwellers, 50.5% were self-employed, and 6.6% had an academic degree. In addition, 30.2 and 2.7% of the participants had a history of smoking and drinking, respectively. In 152 cases (37.1%), the tumor stage was III, and IV in 42 cases (10.2%). Gleason's score of 114 cases (27.8%) was equal to 8 and ≥9 for 117 cases (28.5%). As to the type of treatment, 30 cases (7.3%) did not receive any treatment, 63 cases (15.4%) had active surveillance, 87 cases (21.2%) had radiotherapy, and 86 cases (21%) had orchiectomy (Table 1).

**Table 1:** Demographic and clinical characteristics of PC patients and survival using Kaplan–Meier method

Characteristic	Category	N (%)	Median survival per (sub) category (95% CI)	*P -Value
Age at diagnosis	≤60 years	56 (13.7)	84 (68-98)	<0.001
	61-70 years	150 (36.8)	60 (50-71)	
	71-80 years	143 (34.6)	46 (37-51.5)	
	≥81 years	61 (14.9)	30 (22-34.5)	
Residence	Rural	128 (31.2)	45 (37-50)	0.022
	Urban	282 (68.8)	55 (46-60.5)	
Marital status	Single	30 (7.3)	44 (25-64)	0.327
	Married	380 (92.7)	49 (45-55.5)	
Occupation	Unemployed	107 (26.1)	41 (31.5-50)	0.003
	Worker	62 (15.1)	47 (33-70)	
	Self-employed	207 (50.5)	50 (46-60)	
	Employee	34 (8.3)	62 (51-74)	
Education	Illiterate	141 (34.4)	44 (37-48)	0.002
	Diploma or below	242 (59)	55 (46.5-64)	
	Academic	27 (6.6)	91 (80-102)	
Tobacco history	No	286 (69.8)	48 (44-59.5)	0.978
	Yes	124 (30.2)	49 (42-56)	
Alcohol history	No	399 (97.3)	49 (45-55.5)	0.568
	Yes	11 (2.7)	51 (32.5-68)	
Family history of PC	No	362 (88.3)	50 (46-56)	0.498
	Yes	48 (11.7)	46.5 (30-70.5)	
Comorbidity	No	240 (58.5)	55 (48-63)	0.042
	Yes	170 (41.5)	45 (37-50)	
AJCC stage of disease	I	102 (24.9)	84 (68.5-102)	<0.001
	II	114 (27.8)	70 (61.5-91)	
	III	152 (37.1)	30 (26-35)	
	IV	42 (10.2)	24 (19-26)	
Gleason score	≤ 6	85 (20.7)	102 (91-112)	<0.001
	7	94 (22.9)	61 (60-74)	
	8	114 (27.8)	36.5 (34.5-40)	
	≥ 9	117 (28.5)	19 (17-22)	
Treatment	No treatment	30 (7.3)	31.5 (26-35)	<0.001
	Active surveillance	63 (15.4)	109 (90-123)	
	Radical Prostatectomy	40 (9.8)	102 (87-114)	
	Radiotherapy	87 (21.2)	58 (51.5-64)	
	Radiotherapy + Radical Prostatectomy	51 (12.4)	49 (38-67)	
	Orchiectomy	86 (21)	29 (23-33)	
	Androgen therapy	53 (12.9)	22 (16-26)	

*PC* Prostate cancer, *CI* confidence interval

* Log- rank test – *P* <0.05

**Fig. 1:**
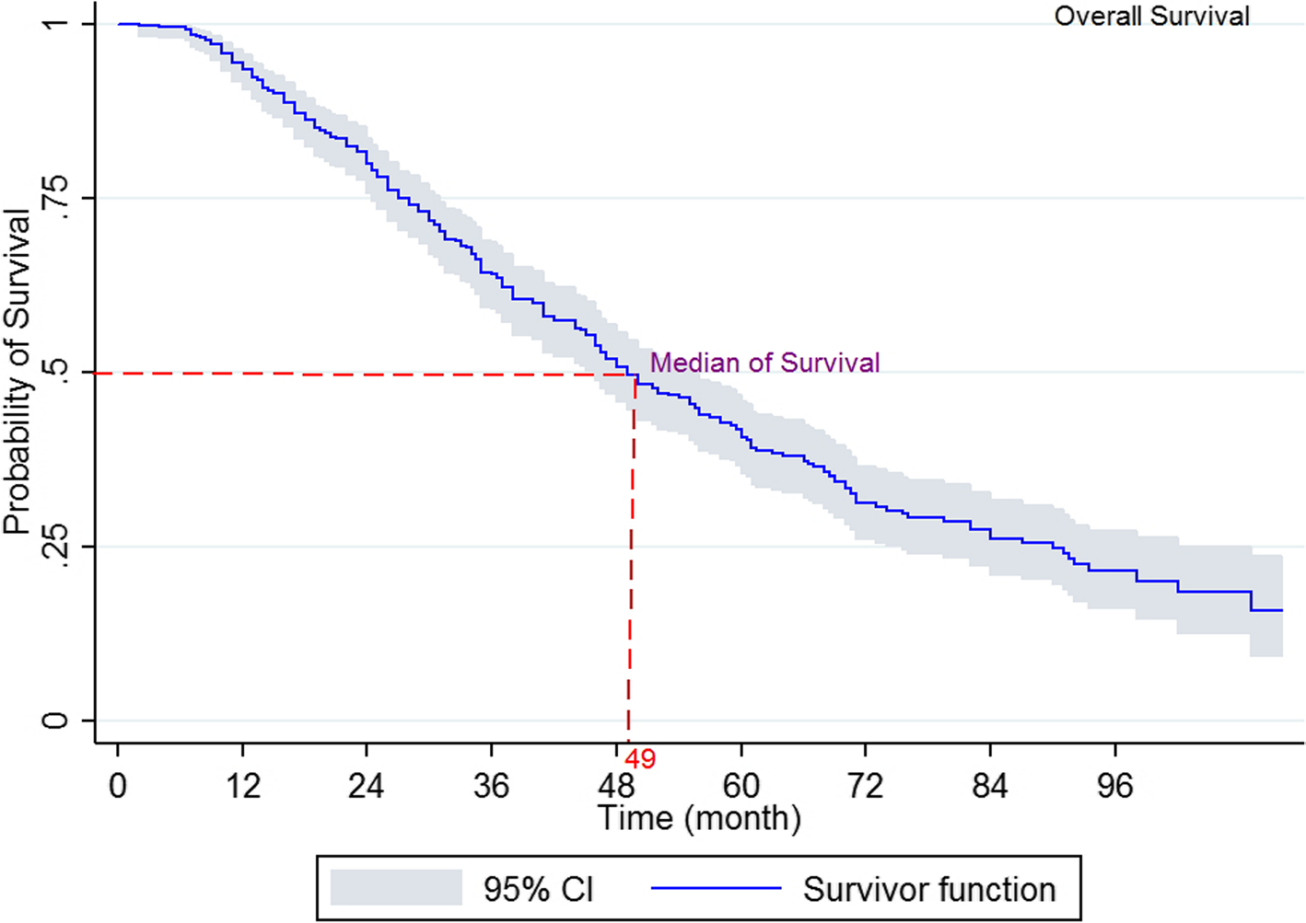
Five-year survival of patients diagnosed with PC cancer in Kurdistan province (2011 - 2018) (Kaplan- Meier). PC= Prostate cancer, CI= confidence interval

**Fig. 2:**
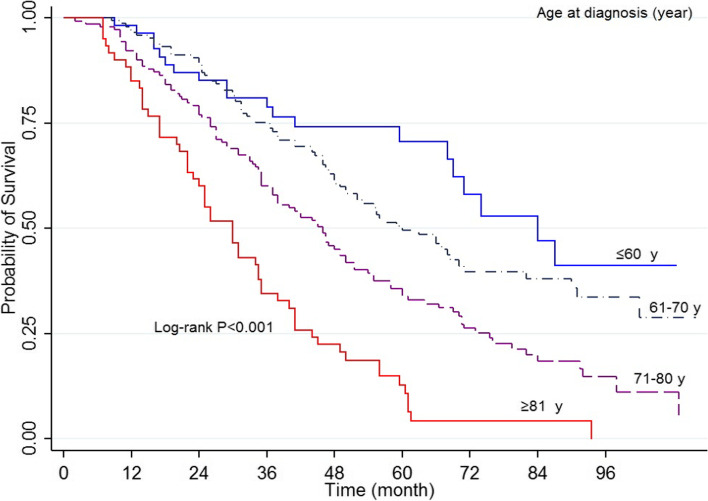
Kaplan–Meier curves of prostate cancer-specific survival across age at diagnosis

**Fig. 3:**
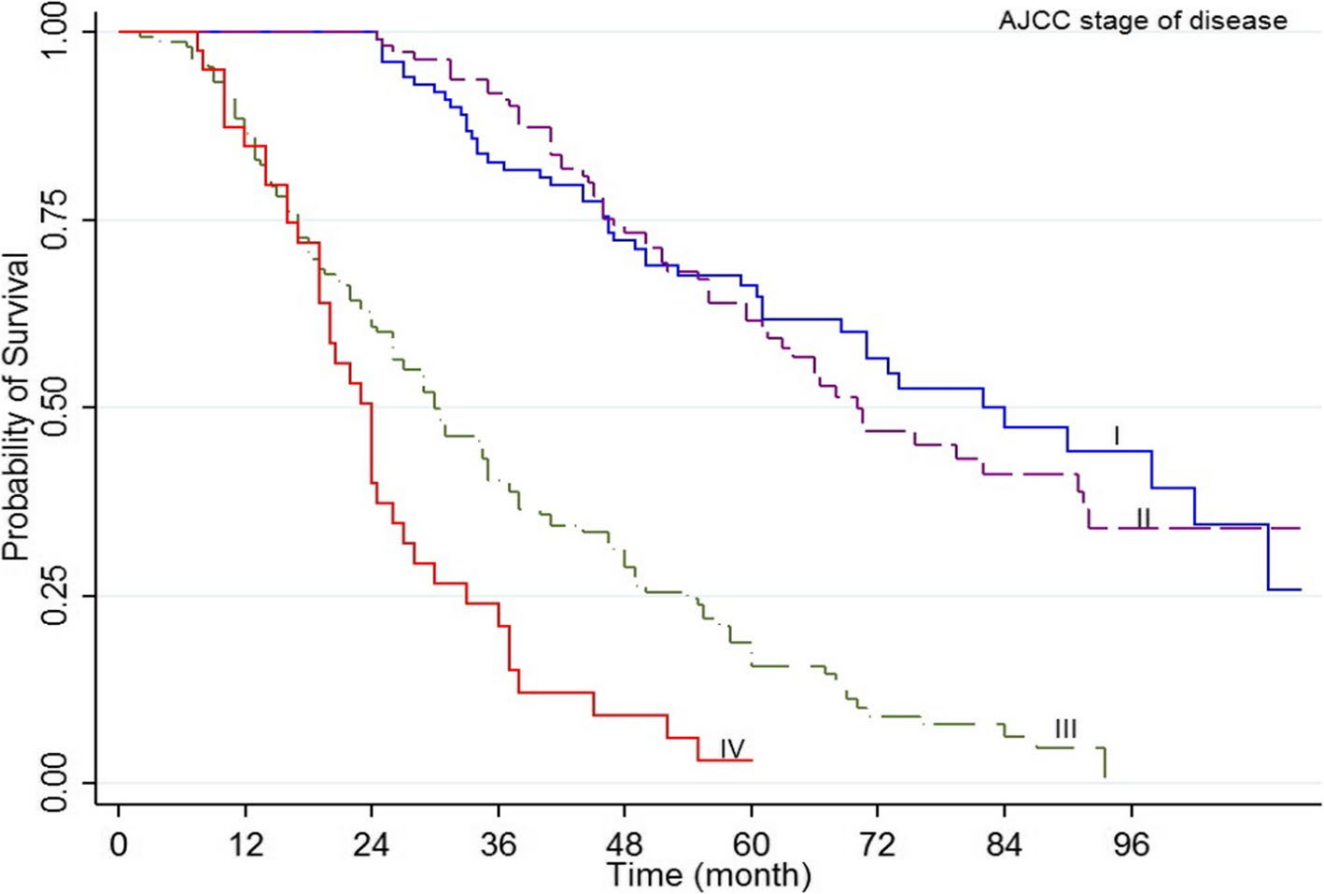
Kaplan–Meier curves of prostate cancer -specific survival across AJCC stage of disease

**Fig. 4:**
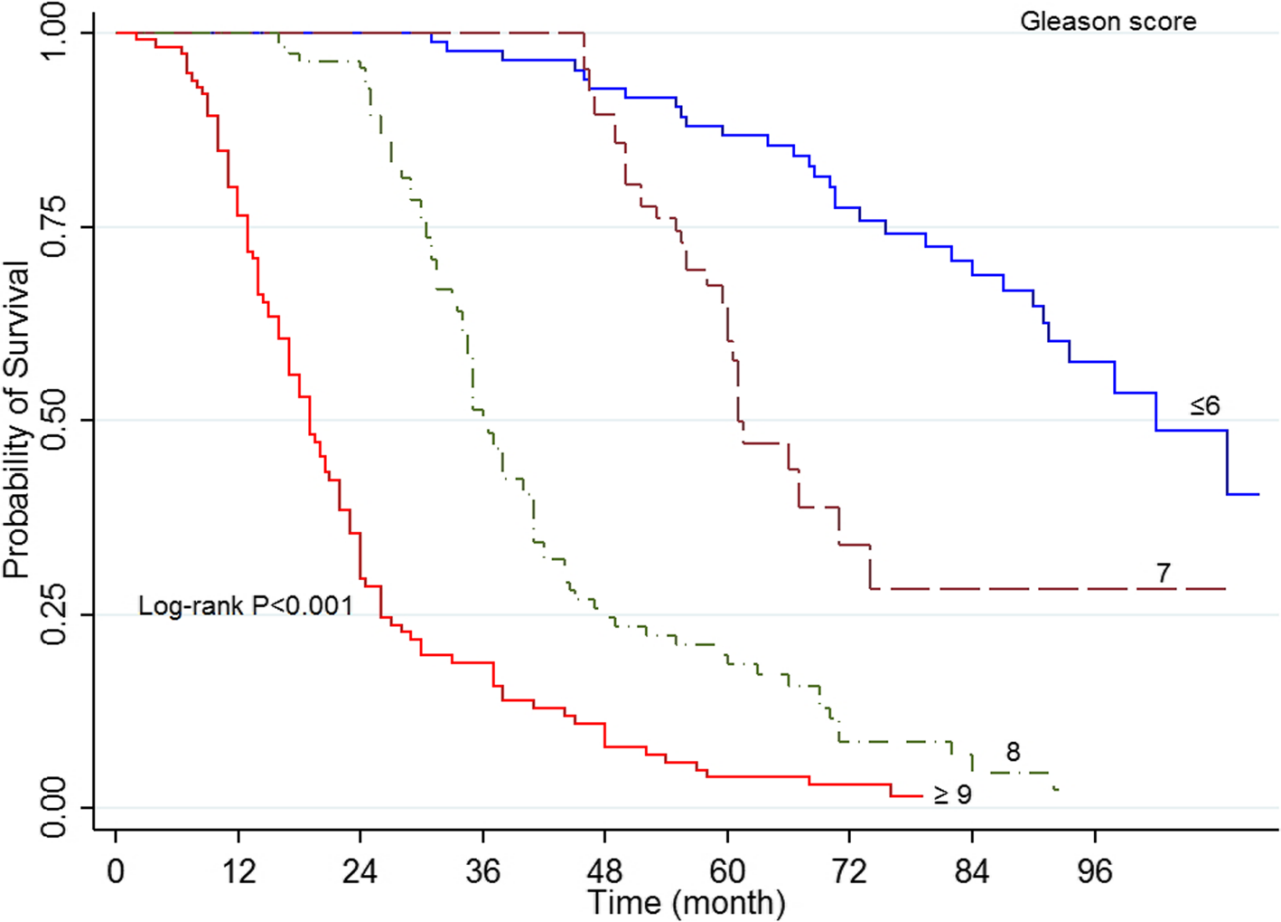
Kaplan–Meier curves of prostate cancer -specific survival across Gleason score

The results indicated that 1, 3, and 5 years’ survival rate was 93, 64.1, and 40.7%, respectively, with mid survival of 49 months (95% CI 55.6-49) (Fig. 1). The log-rank test indicated that the survival rate of prostate cancer was different depending on diagnosis time (*P*<0.001) (Fig. 2), domicile (*P*=0.022), job (*P*=0.003), an education level (*P*=0.002), underlying disease (*P*=042), the position of tumor (*P*<0.001) (Fig. 3), Gleason score (*P*<0.001) (Fig. 4), and type of treatment (*P*<0.001). On the other hand, the survival rate was not significantly related to marital status, history of smoking and drinking, and history of prostate cancer in the family (*P*>0.05) (Table 1).

Multiple Cox regression analysis results showed that individuals older than ≥81-years old had a lower survival rate (HR=2.23, 95% CI: 1.23-4.42, *P*=0.009). In addition, the hazard rate of PC in individuals aged 71-80 years was 1.26 times higher than that of age group younger than ≤60-years old. Univariable regression results indicated that individuals living in the city had a higher survival rate than those living in rural areas (HR=0.74, 95% CI: 0.5-0.97, *P*=0.024). However, multiple regression results indicated no significant difference (*P*=0.067), and the survival rate in individuals with a university degree was higher (HR=0.78, 95% CI: 0.6-0.91, *P*=0.042). Multiple Cox regression results indicated that death hazard in individuals with tumor stage III was equal to 1.45, and that of individuals with tumor state IV was equal to 2.18. The mortality hazard rate in individuals with a Gleason score of 8 was equal to 4.16 (95%CI: 250-6.93, *P*=0.012), and that of individuals with a Gleason score ≥of 9 was 7.14 (95% CI: 5.35-10.28, *P*=0.002). One variable and multivariate regression results showed that individuals who received active care, radical prostatectomy, radiotherapy, combined treatment, and orchiectomy had a lower mortality rate than those who received no treatment. However, those who received ADT treatment did not have a significantly different survival rate (Table 2).

**Table 2:** Univariate and multivariate cox regression analysis for 5-year overall survival rate

Characteristic	Category	Univariate analysis HR (95%CI)	*P -Value	Multivariate analysis HR (95%CI)	*P* -Value
Age at diagnosis	≤60 years	1	-	1	-
	61-70 years	1.47 (0.90-2.42)	0.118	0.86 (0.49-1.50)	0.609
	71-80 years	2.44 (1.57-3.95)	<0.001	1.26 (1.12-2.31)	0.032*
	≥81 years	4.99 (2.98-8.36)	<0.001	2.33 (1.23-4.42)	0.009*
Residence	Rural	1	-	1	-
	Urban	0.74 (0.57.0.96)	0.024	0.98 (0.74–1.23)	0.067
Marital status	Single	1	-	Not in model	-
	Married	0.80 (0.51-1.25)	0.331	-	-
Occupation	Unemployed	1	-	1	-
	Worker	0.80 (0.55-1.17)	0.268	1.09 (0.71–1.68)	0.683
	Self-employed	0.74 (0.55-0.99)	0.043	1.01 (0.74–1.38)	0.967
	Employee	0.28 (0.15-0.52)	<0.001	0.42 (0.20–0.87)	0.021*
Education	Illiterate	1	-	1	-
	Diploma or below	0.83 (0.71-0.93)	0.042	0.91 (0.64–1.11)	0.087
	Academic	0.36 (0.19-0.68)	0.002	0.78 (0.64–0.91)	0.042*
Tobacco history	No	1	-	Not in model	-
	Yes	0.99 (0.76-1.29)	0.978	-	-
Alcohol history	No	1	-	Not in model	
	Yes	0.78 (0.35-1.77)	0.568	-	-
Family history of PC	No	1		Not in model	-
	Yes	1.13 (0.78-1.65)	0.498	-	-
Comorbidity	No	1		1	-
	Yes	1.28 (1.01-1.63)	0.044	1.05 (0.85–1.14)	0.124
AJCC stage of disease	I	1		1	
	II	1.09 (0.74-1.62)	0.634	1.03 (0.61-1.40)	0.733
	III	4.38 (3.09-6.20)	<0.001	1.45 (1.09-2.30)	0.045*
	IV	8.55 (5.41-13.53)	<0.001	2.18 (1.09-3.68)	0.025*
Gleason score	≤ 6	1		1	-
	7	2.19 (1.31-3.63)	0.002	1.21 (0.69–1.40)	0.501
	8	5.32 (2.34-8.97)	<0.001	4.16 (2.50–6.93)	0.012*
	≥ 9	8.14 (5.32-11.64)	<0.001	7.12 (5.35–10.28)	0.002*
Treatment	No treatment	1		1	-
	Active surveillance	0.06 (0.03-0.11)	<0.001	0.14 (0.07-0.29)	0.001*
	Radical Prostatectomy	0.07 (0.03-0.13)	<0.001	0.16 (0.08-0.37)	0.001*
	Radiotherapy	0.25 (0.15-0.40)	<0.001	0.48 (0.28-0.83)	0.008*
	Radiotherapy + Radical Prostatectomy	0.34 (0.20-0.57)	<0.001	0.42 (0.23-0.76)	0.005*
	Orchiectomy	1 (0.64-1.56)	0.977	0.48 (0.29-0.81)	0.006*
	Androgen therapy	0.97 (0.85-1.20)	0.185	0.68 (0.39-1.17)	0.169

## Discussion

The results demonstrated that the age at diagnosis, occupation, education, AJCC stage of disease, Gleason score, and treatment type were influential factors in PC’s survival rate in Kurdistan Province. The survival rate of 1, 3, and 5 years were 93, 64.1, and 40.7%, respectively.

The present study results showed that the mean ± SD age at diagnosis was 68.3±9.82 years, and in a study in Yazd Province-Iran on 113 patients with prostate cancer was 67.3±9.82 years the five-year survival rate was 36.1% [21].

Several studies have been conducted in Asia on the survival of PC. Between 1992 and 2000, PC's relative five-year survival rate in China was 32.5% [22]. However, the five-year survival rate of prostate cancer in South Korea in 1996 and between 2010 and 2014 was 67.2 and 93.3%, respectively, which is ascending [23, 24]. Another study on different ethnic groups in China reported that the survival rate ranged from 26 to 78%, which is a broad-spectrum so that there was a significant difference between different ethnic groups [25]. The survival rate of PC patients has been ascending over the past few years [26].

In all regions, the five-year survival rate increased from 83% in the late 1980s to 99% in late 2008-2014 [27]. According to Colman et al., the five-year survival rate in 31 countries showed a wide range of changes in survival rate in different age groups in different countries. Even after adjusting to cover differences in mortality rate due to other causes, the difference was still considerable in the USA, so the survival rate of Caucasians was higher than Afro-Americans (92.4 vs. 85.8%). The differences can be due to different care qualities and stages of disease [28, 29].

The Steele et al. study showed that the one-year and five-year survival rate was notably higher than previous periods like 2002-2003 and 2004-2009 [30]. Critz et al. reported a 10-year survival rate of prostate cancer in their study in 2013, approximately 75% in the USA [31], which is two times more than what was reported by a meta-analysis study on 11 studies, which was equal to 36.2% [15, 32]. In general, one, five, and ten-year survival rates of PC in Asian countries were less than the world mean rate. In addition, the survival rate was higher in countries with higher HDI [15]. This index is higher for Asian people living outside Asia. Improving survival rate, lead time, over diagnosis, and prostate antigen-specific antigen screening are essential factors. In addition, thanks to new treatments and interventions and an increase in HDI, the survival rate of PC patients has been increasing [27, 32].

PC is not prevalent in men younger than 50 years old. The results indicated a higher mortality rate in individuals diagnosed in 71-80 and above 81 years old. A study in Yazd Province-Iran on 113 patients with prostate cancer showed that 19 patients [21%] were younger than 60 years old. Regression results indicated that the mortality rate in 70-79 age group (HR=3.12, 95% CI:1.12-8.72, *P*=0.029) and older than 80 years old (HR=7.13, 95CI:2.50-20.189, *P*<0.001) was higher [21]. Other studies have shown that most patients with PC who survived were older than 65 years old (82%), while less than 1% were younger than 50 years old [27, 32]. The population-based study in the United States showed that survival PC patients were more deficient among the youngest (40-44 years) and oldest patients. The lower survival among the youngest and oldest age group was mainly due to the degree and stage of the disease [33]. However, the independent effect of age on PC survival has not been well established, and there is some evidence that age is not independently associated with specific cancer survival [34].

In Iran and Kurdistan provinces, since prostate cancer screening is not performed and people are less aware of the signs and symptoms of the disease, so at older ages, they go for diagnosis and treatment, which has a more advanced grade and stage of the tumor.

There was no significant relationship between smoking and drinking history and survival of prostate cancer; socioeconomic condition was not checked in this study. Other studies in Kurdistan Province have demonstrated a higher mortality rate in lower socioeconomic conditions in patients with colorectal cancer [35]. Some studies have argued that the higher mortality rate in lower socioeconomic groups is due to the higher prevalence of co-morbidity in these people [36]. Co-morbidity and other factors can describe this finding. For example, high-risk behaviors such as smoking and drinking are more common in lower socioeconomic groups, partially explaining the lower survival rate in these groups [37, 38]. A study by Xu et al. in China illustrated that there was a significant difference between PC patients with hypertension (28.5%) and a control group (48.3%) in terms of five-year survival rate [39]. Co-morbidity increases the risk of all-cause mortality but not cancer-specific mortality; this may be since co-morbidity increases the risk of death due to non-cancer causes between PC patients. It is evident that co-morbidity influences the survival of patients due to deaths from other causes than cancer and affects the decision-making process of treatment.

The results showed the hazard rate in individuals with a Gleason score of 8 (HR=4.16) and individuals with a Gleason score ≥of 9 (HR=7.12). A study in Iran on people with Gleason scores of 7 and 8-10 reported HR=1.87 (95%CI:1.13-3.11, *P*=0.014) and HR=2.38 (95% CI 1.14-4.98, *P*=0.021) respectively [21]. Gleason’s ranking system for PC measures the invasiveness of cancer. A higher score indicates more invasive cancer and a higher risk of metastasis. It is known that this ranking system is directly related to mortality rate and predicts recurrence after surgery and response to treatment [40].

As the results showed, the HR of the cases with tumor stage III was equal to 1.45, and that of patients with tumor stage IV was equal to 2.18. More than 90% of PC cases are diagnosed in the early stages, so relative five-year survival is close to 100% (tumor staging data are not accurately recorded). However, five-year survival in patients with advanced tumor stage decreased by 30%. The histologic level of PC is essential for prognosis. Therefore, it is recommended to report both Gleason's score and cohort score. The PSA level of the serum completes the clinical examination, TNM and group level, and the AJCC prognostic stage group can be defined [41].

Uni and multivariate regression analyses showed that patients who received active surveillance, radical prostatectomy; radiotherapy; combined treatment; and orchiectomy had a higher survival rate than patients who did not receive any treatment, indicating the effect of treatment on survival. A study on 9772 PC patients between 2010 and 2015 in Iran showed that the patients who had radiotherapy and surgery had 92% five-year survival, while this figure for those without treatment was 67.5% [42].

In the study, Kenrick et al. median overall survival for the total cohort was 25.5 months in black men vs. 21.8 months’ white men (HR=0.81, *P*=0.08) with metastatic castration-resistant prostate cancer. There was prolonged survival in the black population in those who only received hormone-based treatment throughout their treatment course; 39.7 months black vs. 17.1 months white (HR=0.54, *P*=0.019) [43].

The result of a population-based cohort study showed, In age-adjusted and multivariable-adjusted analyses, statin use after ADT was associated with a decreased risk of prostate cancer death (HR=0.82; 0.95% CI 0.69–0.96) [44].

Based on the severity of the disease, some patients might need combined treatments along with radical prostatectomy followed by radiotherapy or ADT with radiotherapy. The type of treatment can be determined depending on age, race, ethnic group, access to oncology services, and socioeconomic condition [45].

Treatment can affect the survival rate of cancer patients, while there is ambiguous evidence about the effect of treatment methods on survival rate. Large-scale trial studies on men with PC in the early stages of PSA showed that the advantages of radical treatment are higher than active surveillance. However, further examinations in different situations did not support such advantages. It is not easy to conclude that treatment affects the survival rate [46, 47].

PC treatment depends on the risk of the progress of the disease, co-morbidity, patient's preferences, and physician's decision. There has been an improvement in the survival rate of PC patients in the UK over the past two decades. Still, survival rate inequality based on socioeconomic has been reported by some studies in the UK, Wales, and Scotland [17].

A meta-analysis study on the survival rate of PC patients in Asia showed that the highest survival rate was in Asian people living in the UK, followed by Japanese. On the other hand, China had the lowest one-year survival rate. Higher HDI is related to a higher survival rate as countries like Japan and Singapore with higher HDI had a higher survival rate. That is not true in India as, although the survival rate is high, HDI in India is lower than that of China [15]. These differences can be explained by differences in the health education program and the quality of diagnostic and treatment services and follow-ups [17].

A wide range of factors can affect prostate cancer survival in less developed regions, such as diagnosis age, stage of the disease, invasiveness of the disease, co-morbidity, and unhealthy habits [48].

Many factors have been investigated for their role in PC survival. Evidence on the role of age and race provided inconsistent results, while socioeconomic status, tumor-related characteristics, and treatment had a main role in PC survival.

Essential factors in the difference in prostate cancer survival rate in Kurdistan province can be the lack of prostate cancer screening, lack of awareness of the symptoms of the disease, late referral of patients (diagnosis of disease in old age), and tumor progression is. Another critical aspect of socioeconomic status is accessing the health care services and quality of testing services available to different socioeconomic groups. These vary in different countries. Also, non-academic education, occupation (worker, farmer), and living in rural areas affect the low survival of prostate cancer in Kurdistan province compared to other regions and countries.

## Conclusion

This study demonstrated that factors such as age at diagnosis, level of education, occupation, AJCC stage of disease, Gleason score, and type of treatments were influential factors in the survival of PC patients in Kurdistan province and needed more attention.

## Data Availability

The datasets used and analyzed during the current study are available from the corresponding author on reasonable request**.**
